# Bone Stress Evaluation with and without Cortical Bone Using Several Dental Restorative Materials Subjected to Impact Load: A Fully 3D Transient Finite-Element Study

**DOI:** 10.3390/ma14195801

**Published:** 2021-10-04

**Authors:** Raul Medina-Galvez, Oriol Cantó-Navés, Xavier Marimon, Miguel Cerrolaza, Miquel Ferrer, Josep Cabratosa-Termes

**Affiliations:** 1 Faculty of Dentistry, Universitat Internacional de Catalunya (UIC), 08017 Barcelona, Spain; ruldoc@uic.es (R.M.-G.); oriolcanto@uic.es (O.C.-N.); cabratosa@uic.es (J.C.-T.); 2 Bioengineering Institute of Technology, Universitat Internacional de Catalunya (UIC), 08190 Barcelona, Spain; mcerrolaza@uic.es; 3 Automatic Control Department, Universitat Politècnica de Catalunya (UPC-BarcelonaTECH), 08034 Barcelona, Spain; 4 School of Engineering, Science & Technology, Valencian International University, 46002 Valencia, Spain; 5 Department of Strength of Materials and Structural Engineering, Universitat Politècnica de Catalunya (UPC-BarcelonaTECH), 08034 Barcelona, Spain; miquel.ferrer@upc.edu

**Keywords:** FEA, FEM, impact test, transient analysis, dynamical forces, biomechanical behavior, implant rehabilitation, rehabilitation materials, crown materials, bone loss

## Abstract

**Statement of problem**. Previous peri-implantitis, peri-implant bone regeneration, or immediate implant placement postextraction may be responsible for the absence of cortical bone. Single crown materials are then relevant when dynamic forces are transferred into bone tissue and, therefore, the presence (or absence) of cortical bone can affect the long-term survival of the implant. **Purpose**: the purpose of this study is to assess the biomechanical response of dental rehabilitation when selecting different crown materials in models with and without cortical bone. **Methods**: several crown materials were considered for modeling six types of crown rehabilitation: full metal (MET), metal-ceramic (MCER), metal-composite (MCOM), peek-composite (PKCOM), carbon fiber-composite (FCOM), and carbon fiber-ceramic (FCCER). An impact-load dynamic finite-element analysis was carried out on all the 3D models of crowns mentioned above to assess their mechanical behavior against dynamic excitation. Implant-crown rehabilitation models with and without cortical bone were analyzed to compare how the load-impact actions affect both type of models. **Results**: numerical simulation results showed important differences in bone tissue stresses. The results show that flexible restorative materials reduce the stress on the bone and would be especially recommendable in the absence of cortical bone. **Conclusions**: this study demonstrated that more stress is transferred to the bone when stiffer materials (metal and/or ceramic) are used in implant supported rehabilitations; conversely, more flexible materials transfer less stress to the implant connection. Also, in implant-supported rehabilitations, more stress is transferred to the bone by dynamic forces when cortical bone is absent.

## 1. Introduction

The quantity and quality of bone tissue around a rehabilitated implant play a key role in its long-term survival [1]. After peri-implantitis, immediate implant placement, or regenerated bone, cortical bone is missing for a variable period [2]. Moreover, the absence of cortical bone can affect the biomechanical behavior of both the bone tissue and its ability to withstand impact loads [3].

Different models and techniques are used to analyze the behavior of dental implants [4] including two-dimensional (2D) or three-dimensional (3D) finite element analyses (FEA), photo-elastic studies, or digital image-correlations (DIC). Also, ultrasonic wave analysis [5,6] can be used successfully in dental implant analysis [7].

FEA, whether in 2D or 3D, is a numerical and approximate technique that yields results depending on both the geometry and mechanical properties of the materials. Regarding photo-elastic techniques, when either static or dynamic forces are applied, isochromatic fringes appear in photo-elastic studies [8], thereby allowing the stress distribution at implants to be calculated. The DIC is an image-based analysis that shows how points inside resin blocks move when static or dynamic forces are applied [9]. On the other hand, the analysis of implant behavior requires that the difference between static forces (due to clenching) and dynamic forces (due to mastication or eccentric bruxism) be very clear, particularly when selecting the rehabilitation materials [10,11].

Other works reported similar results for implant behavior [4,8]. These authors showed that the main differences are due to variables such as the type of implant connection, the diameter/length of the implant, the type of rehabilitation material or whether the load was applied statically or dynamically [8,12]. However, the presence or absence of cortical bone is an issue that, to date, was not studied or quantified sufficiently. The absence of cortical bone can lead to the implant loosening and eventually to implant failure [13,14,15,16,17].

From a mechanical perspective, and according to some previous works [18,19,20,21,22], static analysis is not completely enough to get precise and reliable results. Several authors agreed that it is needed to perform dynamic analysis. Dynamic analysis can be found in literature [18,22] but addressing fatigue analysis and not impact dynamic loading.

In previous works [23] the authors analyzed the mechanical behavior of implants with different restorative materials subjected to static loads by three different methods: finite element method (FEM), digital photoelasticity (DP), and digital image correlation (DIC). They concluded that (1) all 3 methods provide very close solutions; (2) FEM is enough reliable and robust for predicting the tooth-implant mechanical behavior, and (3) dynamic impact analysis is mandatory for getting more accurate and closer results to the problem’s physical reality.

Therefore, in this work a fully 3D dynamic FEA study of the influence of different crown materials on the implant behavior with and without cortical bone when subjected to impact dynamic loads is performed. Moreover, we also discuss two clinical cases: (a) both trabecular and cortical bone, and (b) only trabecular bone.

Significant differences in the mechanical response when the peri-implantitis generated a loss of cortical bone were encountered. The study concludes with a comparative dynamic analysis of crowns made with different restorative materials.

## 2. Materials and Methods

### 2.1. The Model

**Figure 1:**
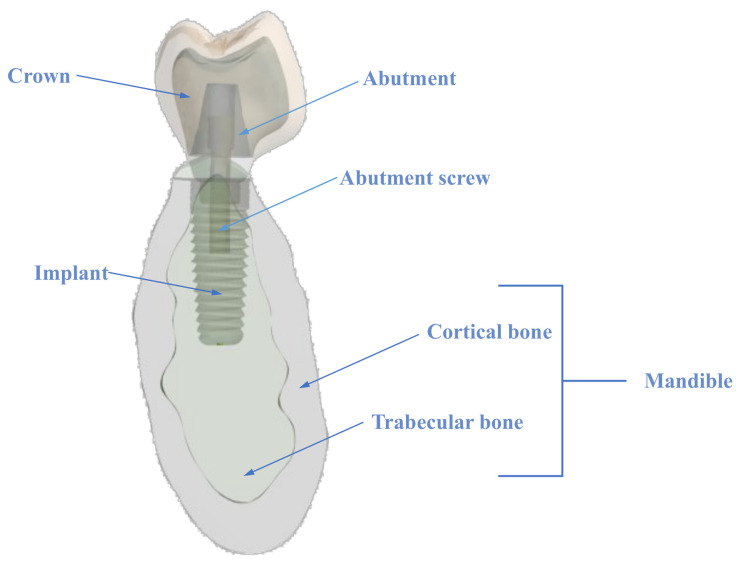
Cross-sectional view of 3D model.

A 3D dynamic finite-element model comprising a crown, an abutment screw, an abutment, an implant, and the surrounding bone was set up to evaluate the von Mises stresses at both the cortical and trabecular bone (See Figure 1). Ethics approval was not required for this in-vitro study. All the parts of the model are described hereafter.

#### 2.1.1. The Crown

**Figure 2:**
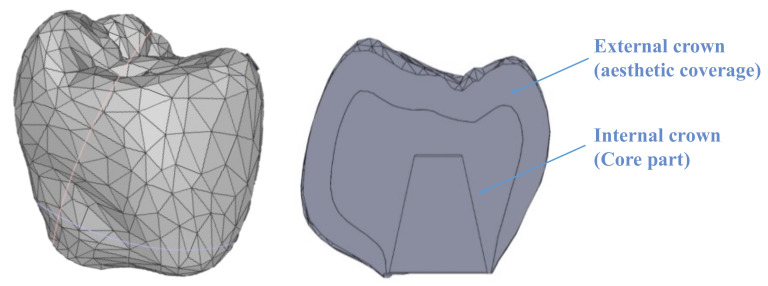
Crown model. Left: cross section showing both internal and external crown.

The crown model was processed using Solidworks (version 2021, Dassault Systèmes, Waltham, MA, USA) [24] by importing a CAD model generated by Exocad-3D (v3.0, Exocad GmbH, Darmstadt, Germany). The model was built by assembling two components: the core (51% of the total volume) and the aesthetic veneering (40% of the total volume), as displayed in Figure 2. The volume of the resulting crown model is 411.5 mm^3^.

#### 2.1.2. The Abutment and Screw

The abutment is a metal component whose function is to attach the crown and the implant. Also, a hexagonal antirotation system prevents the relative movement between the abutment and the implant. The screw allows the connection between the abutment and the implant.

**Figure 3:**
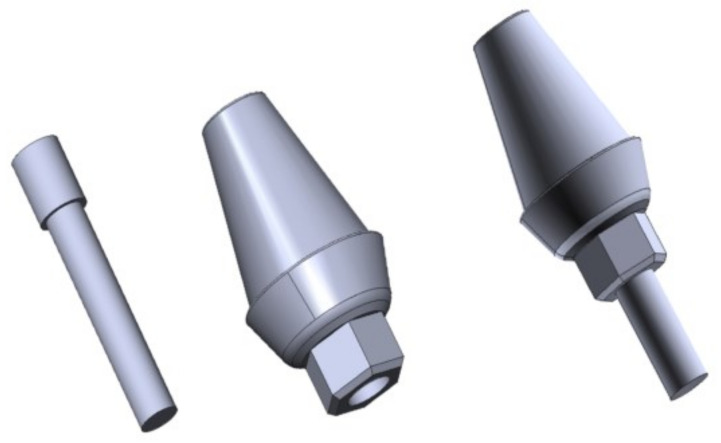
Left: abutment screw; center: abutment with hexagonal antirotational system; right: screw-abutment assembly.

Solidworks was also used to model the abutment and the screw, thus reducing the classical surfaces to a simpler triangular shapes (see Figure 3). The model corresponds to the hexagonal-connection abutment of the MIS implant [25].

#### 2.1.3. The Implant

**Figure 4:**
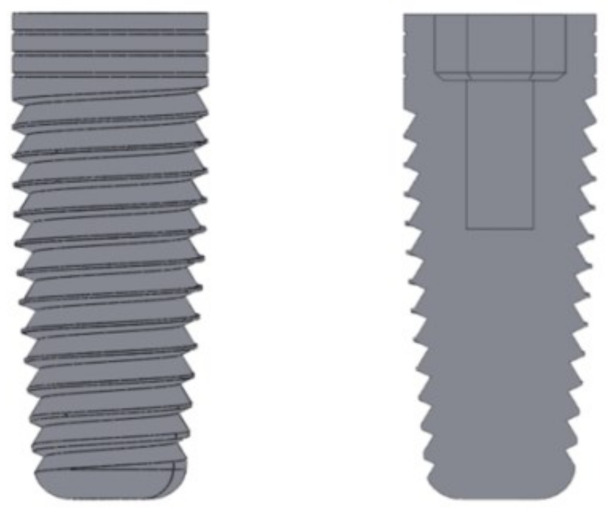
Implant. Left: lateral view; right: cross section.

Figure 4 displays the implant created with Solidworks to obtain more accurate geometric shapes. The implant used in this study was a 4.2 × 11.5 mm^2^ MIS implant [25], because it is a well-known and reliable implant used in the area of restorative dentistry and world-wide commercialized.

#### 2.1.4. The Mandible

**Figure 5:**
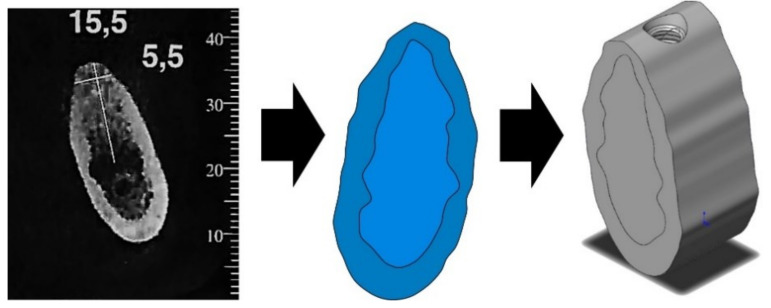
Left: CT scan. Center: mandible section obtained from CT scan. Right: isometric view of mandible’s model.

The mandible section was made from a CT scan section [26]. The mandible’s geometry was generated by following two steps: (a) some key points were defined at a known distance at the CT-scan section, and (b) the scan scale was then used to compute the actual distances at the real mandible. The 3D geometry of the mandible was split into an external region of cortical bone and an internal one of trabecular bone, each one with its specific mechanical properties (see Figure 5).

#### 2.1.5. The Loading Plate

**Figure 6:**
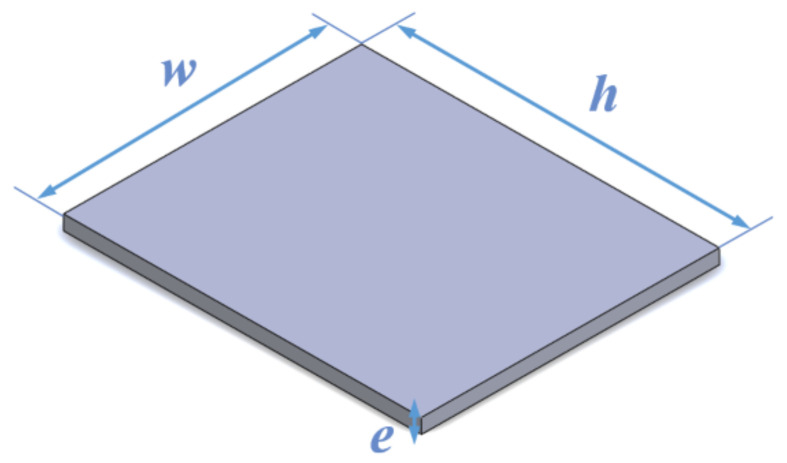
Dimensions of rigid plate (*w* = 10 mm, *h* = 12 mm, *e* = 2 mm).

The impact loads produced by chewing were applied to the model (crown, implant, and mandible) by using a rectangular rigid plate dimensioned as shown in Figure 6. Some boundary conditions were considered: (a) the initial separation between the plate and the crown was set to 0.01 mm; (b) free sliding is assumed, i.e., the impact of the plate on the model is frictionless; (c) normal stresses were considered null if the plate loses contact with the model.

### 2.2. Geometry of the Assembled Model

**Figure 7:**
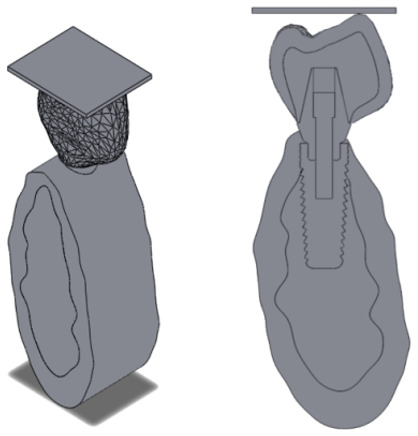
Assembled geometry. **Left**: isometric view; **right**: cross section.

After all the individual parts were independently built, they were assembled together as shown in Figure 7.

Before conducting the FEA simulation, the assembled geometry was converted to IGES format, which was imported into Ansys Workbench Design Modeler (version 2021, ANSYS, Inc, Canonsburg, PA, USA). Perfect osseointegration between the implant and the bone was assumed. This means that the bone is integrated into all slots of the placed implant, although this does not always happen in clinical cases

### 2.3. Materials of the Model

The mechanical properties of the materials have a direct influence on the calculation of the stress response and its deformations. All materials were considered elastic, homogeneous, and isotropic. Mandible bone mechanical properties can be found elsewhere [27]. Different materials were used in the crown, to discover if the crown material has a direct effect on the implant stress distribution and mechanical performance (see Table 1 below):

A total of six crown rehabilitation materials were analyzed: the carbon fiber-composite crown (FCOM) had an aesthetic layer of a BioXfill composite [29] and carbon fiber framework of BioCarbon Bridge; the metal-ceramic crown (MCER) had a framework of LaserPFM Co-Cr metal alloy and aesthetic veneering of VITA VMK 95 ceramic material [30,31]; the metal-composite crown (MCOM) had a framework of LaserPFM Co-Cr metal alloy [30] and BioXfill composite [29]; the metal crown (MET) is made entirely of melted Co-Cr alloy [32]; the carbon fiber-ceramic crown (FCCER) had a framework of BioCarbon Bridge carbon fibers [29] and lithium disilicate ceramic aesthetic covering [33]; finally, the PEEK-composite crown (PKCOM) is made in the inner part of polymer polyetheretherketone (PEEK) and the aesthetic veneering with BioXfill composite [34].

All the mechanical properties of each crown, the implant, and both bone tissues (cortical and trabecular) used in the numerical simulation are listed in Table 1. The mechanical properties of the materials were obtained from the manufacturer datasheets indicated in the table.

### 2.4. Numerical Simulation

Although there are numerous studies centered on static forces, there are few studies of the dynamic forces applied during physiological functions. Some biomechanical estimated values can be obtained in the literature [35,36]. Therefore, a simulated impact between the rigid plate and the whole implant geometry was carried out. **Results were analyzed over time due to the dynamic response of the model**. The simulation was performed with ANSYS (version 2021, ANSYS, Inc, Canonsburg, PA, USA) [37] using the mechanical APDL solver and the Transient Structural analysis system.

#### 2.4.1. Simulation Setup

A first simulation was performed to check that all the materials, hypotheses, and boundary conditions of the model were correctly defined. All the results were coherent, but the computational time was very high for two main reasons: there was a very large number of nodes and elements and the time set for analysis was more than was needed. Therefore, the time and number of elements and nodes were reduced. The distance between the plate and the crown was also shortened. Furthermore, to carry out reasonable comparisons, the dynamic characteristics of the impact, i.e., initial kinetic energy and linear momentum, should be the same in all the simulations.

Initially, all the mass particles of the dental implant have the same velocity as well as the kinetic energy due to the movement of the whole structure (mandible, implant, crown, and abutment), as shown below:

(1) $$E_{k}=\sum \frac{1}{2}\cdot m_{i}\cdot v^{2}$$

where *E_k_* is the kinetic energy, *m_i_* is the particle mass, *v_i_* is the particle velocity.

When the dental implant collides with the plate the kinetic energy is transformed into strain energy. The strain energy for a deformable solid can be determined through the stress and strain tensors time-history.

If the deformation occurs within the linear-elastic range, the potential elastic deformation energy can be obtained from Equation (2):

(2) $$E_{def}=\int_{V}^{}\sigma_{i}\cdot \varepsilon_{i}\cdot dV$$

where *E_def_* is the potential elastic deformation energy, *σ_i_* is the stress state, *ε_i_* is the strain state, *dV* is the volume differential.

The dynamic characteristics of an impact are determined by the system’s initial energy (0.5·m·v^2^) and linear momentum (m·v). Both features must have the same value in any case, which is guaranteed by introducing the same speed (v) and the same mass (m). On the other hand, the effect of the mandible’s mass is not so significant because it is externally fixed.

Since each crown material has a different density, the initial mass of the system and, consequently, its initial kinetic energy would be different for every combination case. Then, the base mass (implant + mandible) was leveled in all models to balance the crown’s mass changes. Note that the base mass represents the whole mass of the mandible, which should be assumed to be the same in all cases. Therefore, a uniformly distributed mass was added to the models to make the impacts equivalent energetically. Table 2 shows the additional mass added to each model.

Two changes were made to the whole model to simulate the absence of cortical bone, as in peri-implantitis, peri-implant bone regeneration, or immediate implant placement. Firstly, the cortical part of the assembly was suppressed, and the rest of the mesh is kept intact. Therefore, the nodes chosen (see section ‘Node selection’) are the same and can be compared. Secondly, some mass was added to equal the impact energy. This added mass corresponds to the suppressed cortical bone and is obtained by multiplying the volume by its density. It is constant for all models, and the value is 2.27 g. Table 3 lists the added mass in the lateral surface for each crown material:

#### 2.4.2. Finite Element Mesh

Now, the next step is to generate a suitable finite element mesh, which must be done carefully since the precision of the results depends largely on how refined the mesh is. Then, if greater precision is needed, smaller elements should be used. The more refined the mesh, the more computer time is required. This does not have a large impact in finite element (FE) static analysis but, nevertheless, it is a key aspect in FE transient dynamic analysis.

**Figure 8:**
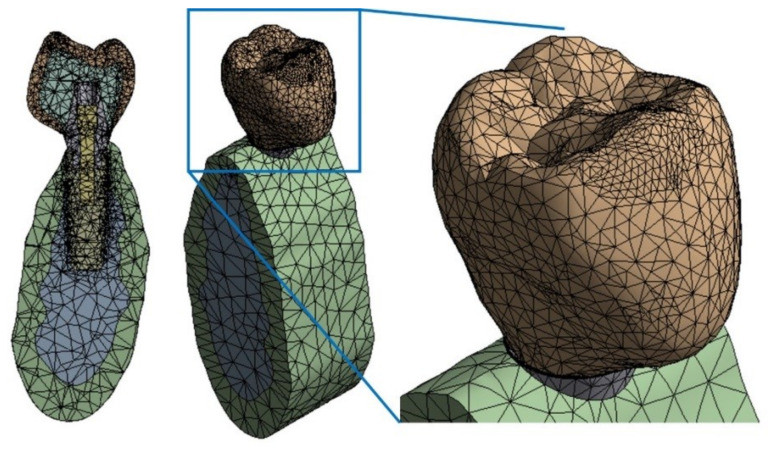
Model mesh (96,160 nodes and 62,606 elements). **Left**: cross section; **center**: isometric view; **right**: details on top of crown.

A mesh sensitivity analysis was done to ensure a reliable model mesh. The refining process started from an initial mesh of 57,330 nodes until reaching a final mesh of 96,160 nodes, where the difference between stress values at some selected nodes did not exceed 5%. Figure 8 shows the finite elements mesh.

The Ansys 3D solid element type SOLID187 was used, with 10 nodes and quadratic interpolation functions, which fits better to irregular geometries. The element has three degrees of freedom per node, i.e., the three translations in the global coordinate directions *x*, *y*, *z*. The surface-to-surface contact was defined with the element CONTA174.

Also, a refinement was set for the impact zone, thereby ensuring better accuracy in this area. Therefore, as this refinement was done, it is not necessary to use an area to obtain an average solution, since the nodal solution is especially accurate. In addition, Ansys software performs a control of the aspect ratio systematically.

The size of the finite element mesh, related to its accuracy, is smaller at the loading point and the threaded part (0.2 mm), where higher accuracy is needed, but larger in the other parts (from 0.5 to 2 mm). To find the optimal mesh and an acceptable computational time, a sensitivity analysis of the mesh was also carried out. First of all, a coarse mesh was used, and subsequently, finer meshes were tested. The refining process was stopped when results became stabilized.

#### 2.4.3. Initial and Boundary Conditions

In dynamic analysis, the boundary conditions (BC) are displacements, velocities, and accelerations instead of static forces and displacements as in static analysis. There are many publications and studies offering strategies on how to configure the loads to obtain a realistic simulation in static analysis. But in dynamic analysis the loads are the result of the calculation, i.e., the input for an impact simulation is the model’s initial energy, not an applied load.

Energy concepts allow us to relate the model mass (bone-implant-abutment-crown) and the velocity applied as an initial boundary condition. The mass was calculated according to a density and volume of 411.5 mm^3^ for each crown (see Table 1). The model was also subjected to an initial velocity of 1 m/s as the initial kinematic condition.

Some studies focus on the mandibular kinematics by using small accelerometers [33]. The peak peripheral acceleration when opening the mouth can reach an average of 2.5 m/s^2^. Considering this and keeping in mind that we are dealing with a brain-controlled occlusally system, the same acceleration (*a*) during occlusion can be assumed. An estimated velocity (*v*) can now be obtained using this value and the formula of the constant acceleration movement:

(3) 1v=a⋅Δt

where, *v* is the velocity, *a* is the acceleration, *Δt* is the time increment.

Considering that the mastication occlusion lasts less than 0.5 s, according to Equation (3) we can write: *v* = *a*·Δ*t* = 2.5·0.5 = 1.25 m/s. Therefore, a reasonable value for the velocity condition would be 1.25 m/s.

Note that the bottom surface of the plate was fixed. This means that it cannot move or rotate in any direction. During the impact, certain forces appear to counteract the vertical displacement of the rest of the bodies. The distance between the plate and the external crown is 0.01 mm. The lateral surfaces of the cortical and trabecular bones were fixed in the normal (*z*-axis) and tangential (*x*-axis) directions, thereby having a degree of freedom in the direction of the displacement (*y*-axis). As a result, the bones only move in the direction of the vertical axis and do not rotate in any direction.

#### 2.4.4. Simulation Time

The simulation time-limit also has to be set. The impacts happen in a very short period. Some previous works in other scientific fields found that the impact lasts less than one millisecond [38,39], but there is very little (or almost no) published work on dynamic impact loads in dental applications. On the one hand, the duration of the simulations should be as short as possible to reduce the CPU computation time; but on the other, it should be longer than the impact duration and its further effects. Finally, the simulation time was set at 0.4 ms.

Now, the time interval between two analysis steps (known as time-step or Δ*t*) needs to be defined. Similar to what was discussed about mesh’s size, the shorter the time-step, the more accurate the results and the more computer time is required. After some sensibility numerical analyses, a time-step of Δ*t* = 7.55 µs was found to lead to sufficiently accurate results.

#### 2.4.5. Node Selection

**Figure 9:**
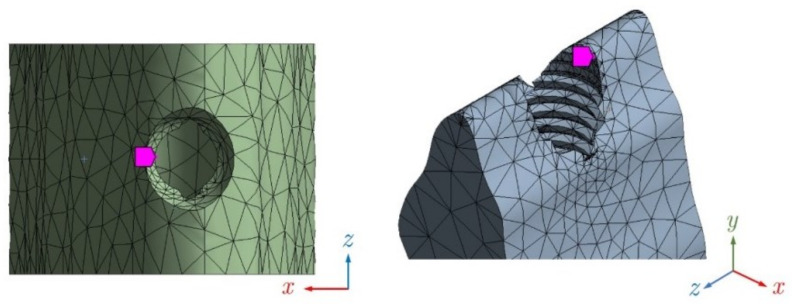
Benchmark nodes shown with a purple marker. **Left**: node at top of cortical bone; **right**: node at top of trabecular bone.

To conduct this study, the energy of the object was computed over two benchmark nodes: one at the top of the cortical bone, and the other at the top of the trabecular bone (see Figure 9). The dynamic simulation was run on the six types of crown, with and without cortical bone. As the mesh is the same for all the models, the selected nodes are also the same.

Two simulations were performed. In the first simulation, a healthy bone was considered, with a cortical and trabecular part, where the stresses at the cortical node and the trabecular node were measured. In the second simulation, a cortical bone absence was considered; this is only containing trabecular part, so stresses were measured only at the trabecular node (See Table 4).

## 3. Results

The von Mises stress value was calculated and compared for each node. Table 5 shows the values of maximum von Mises stress obtained over time in the dynamic FEA simulation, with cortical bone at both nodes (cortical and trabecular) and the trabecular node without cortical bone.

**Figure 10:**
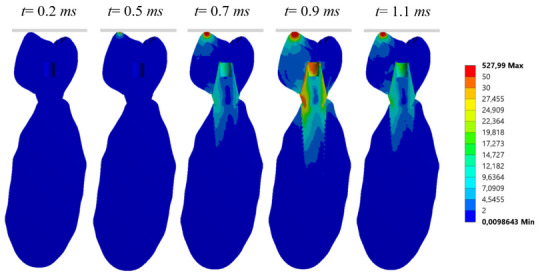
Von Mises stress distribution over time at a cross section using the Carbon Fiber-Ceramic crown (FCCER), considering both trabecular and cortical bone.

The von Mises stresses increase of trabecular bone without cortical with respect to the trabecular bone with cortical were calculated as follows:

(4) $$\Delta \sigma_{\mathrm{vM}}(\%)=\frac{\sigma_{\mathrm{vM}}{\;}_{\mathrm{trab}+\mathrm{cort}}-\sigma_{\mathrm{vM}}{}_{\;\mathrm{trab}}}{\sigma_{\mathrm{vM}}{}_{\;\mathrm{trab}+\mathrm{cort}}}\cdot 100$$

where σ_vM_ is the von Mises stress. The distribution of stress varies according to the time, reaching the maximum values of stress during the impact (see Figure 10).

### 3.1. Cortical Node (with Cortical and Trabecular Bone)

**Figure 11:**
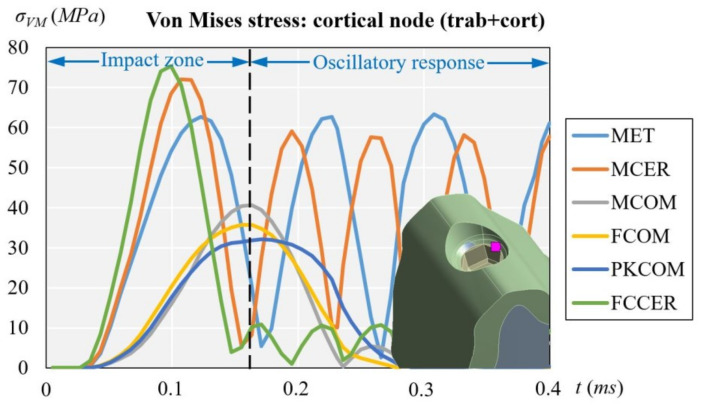
Comparison of equivalent von Misses stress over time on all crown materials at cortical node, considering both trabecular and cortical bone.

Figure 11 shows the evolution of the von Mises stress of the dynamic simulation over time for the different models at the cortical node. The results obtained at the cortical node were (units in MPa): 32.05 (PKCOM), 35.70 (FCOM), 40.71 (MCOM), 63.35 (MET), 72.06 (MCER), and 75.46 (FCCER).

### 3.2. Trabecular Node (with Cortical and Trabecular Bone)

**Figure 12:**
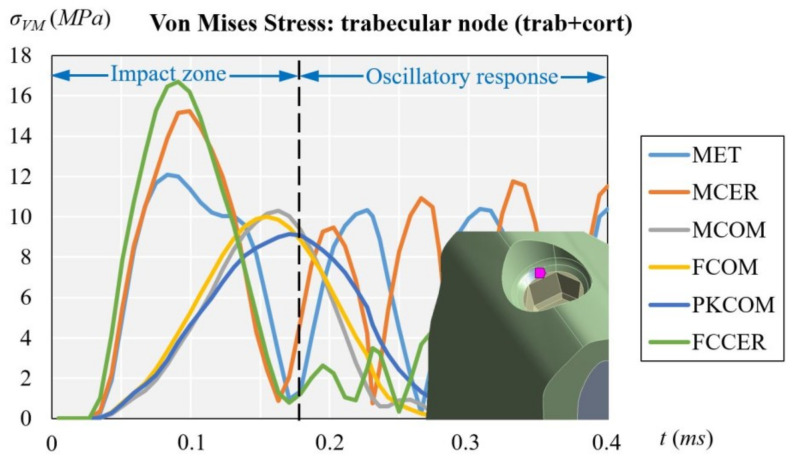
Comparison of equivalent von Mises stress over time depending on all crown materials at trabecular node, considering both trabecular and cortical bone.

Figure 12 displays the evolution of the von Mises stress of the dynamic simulation over time for the different models at the trabecular node. The results obtained at the trabecular node (units in MPa) were: 9.15 (PKCOM), 10.01 (FCOM), 10.30 (MCOM), 12.09 (MET), 15.23 (MCER), and 16.69 (FCCER).

The evolution of the stress in the trabecular node displays the same pattern as in the cortical node but the stress values are considerably lower, since the trabecular bone is not very dense and is less rigid than cortical bone. Moreover, the trabecular bone receives the effects of the impact-load later. The shape of the function is almost equal to that of the cortical node, with more irregular oscillations at the end. Similar as the case of cortical node the maximum stress is in FCCER.

Figure 11 (cortical node) and Figure 12 (trabecular node) show the high peak of stress and the duration over time for FCCER and MCER, which were much longer than for the composite-coated crowns, MCOM, FCOM, and PKCOM. The stress fluctuations due to the eccentricity of the impact load with respect to the implant longitudinal axis can also be observed. The maximum stress values in the ceramic models (MCER and FCCER) are notably higher than in the rest of the models.

### 3.3. Trabecular Node (with Trabecular Bone Only)

**Figure 13:**
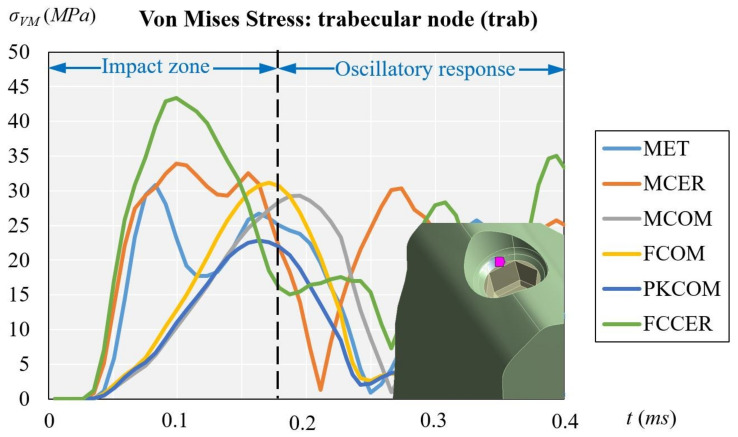
Comparison of equivalent von Mises stress over time at trabecular node, considering trabecular bone only.

This section is devoted to analyzing the effects of the pathological bone without cortical bone (peri-implantitis, bone regeneration, or immediate implant placement). Figure 13 displays the evolution of the von Mises stress of the dynamic simulation over time for the different models at the trabecular node. The results obtained trabecular node (units in *MPa*) were: 22.80 (PKCOM), 31.14 (FCOM), 29.30 (MCOM), 30.80 (MET), 33.87 (MCER), and 43.33 (FCCER). Figure 12 shows, like Figure 11, a similar behavior of the oscillations after the first peak happens. In Figure 13 the frequency of the oscillations is lower as a result of a more damped impact. Results at the cortical node (see Figure 11) showed that the behavior of the stress in the trabecular node is the same as the cortical but the values are much lower. Since the trabecular bone is not very dense, it is more flexible and works as a stress reducer.

**Figure 14:**
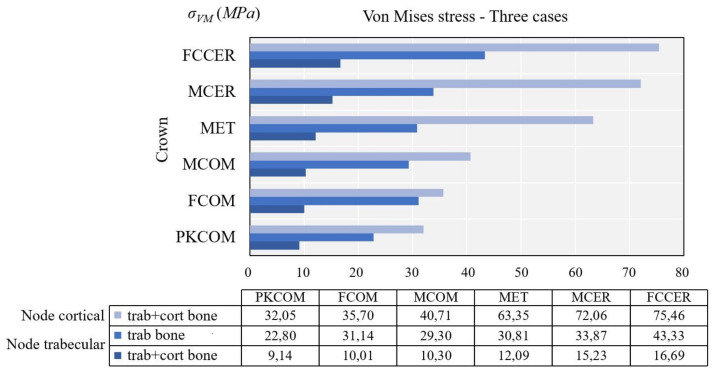
Comparison of maximum von Mises stress obtained at different nodes.

Figure 14 shows a comparison between the maximum von Mises stress obtained in trabecular node with and without cortical bone.

Figure 14 also shows a significant decrease of the von Mises equivalent stress at the trabecular bone with cortical compared to the case without cortical bone. The reason is that in the case with cortical bone, the impact was affecting the whole jawbone and cortical and trabecular bones, so the stress was divided into the two parts. The biggest increase in absolute value was observed in the MCER, with approximately 19 MPa difference.

## 4. Discussion

This study focused on comparing the stresses generated on cortical bone using six types of crown materials placed on a specific implant under impact-load conditions, so the same implant was maintained in all simulations. Therefore, the same methodology can be used to analyze the dynamic response of other implant models.

Since the forces applied in the masticatory process and eccentric bruxism are dynamic, it is important not only to study the mechanical behavior of the restorative materials in static forces, but also to know the mechanical behavior produced by dynamic forces. The rehabilitation materials used in the oral cavity could absorb impacts that may vary according to their mechanical properties [10,40,41,42]. Therefore, the evaluation of the mechanical behavior of the most widely-used materials in implant supported rehabilitations becomes a key issue. Moreover, the morphological, elemental, and biochemical structure without cortical bone is different to healthy bone structure [2].

Figure 11 and Figure 12 (healthy bone) show that over time the reaction of the crown veneered with ceramic was different to that of the crown veneered with composite or with peek as a core. Many fluctuations in the MCER can be observed over time, and the results of the rebound produced by the material’s stiffness, whereas in the crowns with less rigid materials (MCOM, PKCOM, or FCOM), only one fluctuation was observed.

Figure 13 (trabecular node: only trab.) does not have the damping effect of Figure 11 (cortical node: cort. and trab. bone) and Figure 12 (trabecular node: cort. and trab. bone). On the other hand, Figure 11 and Figure 12 can be analyzed through the type of bone they represent, although the shape and location of the oscillations are similar, the Figure 12 has lower amplitudes.

The implant-crown rehabilitation materials made from a combination of metals and ceramics (FCCER, MET, MCER) are more rigid. In those materials, an initial stress peak of higher magnitude is observed, as compared to polymeric materials (PKCOM) that are less rigid. On the other hand, FCOM and PKCOM composites (less rigid) presented very low oscillations which were quickly damped.

It is important to transfer less stress to bone in cases of immediate implant placement, bone regeneration, or after peri-implantitis healing, given that there is no bone for some time in those cases. Hence, using materials that transfer less stress to the bone could avoid problems. In short, crowns made of rigid materials present a greater risk over time of bone loss around implants in the presence of gingival inflammation, since these materials transfer more stress to the bone, whether cortical or trabecular.

## 5. Conclusions

Our study demonstrated that more stress is transferred to the bone when stiffer materials (metal and/or ceramic) are used in implant supported rehabilitations and, conversely, more flexible materials transfer less stress to the implant connection. These relationships between materials’ elastic properties and the dynamic force transmission are in accordance with the findings of different authors [10,40,41,42,43].

The presence (or absence) of cortical bone around the implant connection generates different behavior in rehabilitation implants when dynamic forces are applied, such as grinding, swallowing, or eccentric movements. Such transferred force, on the other hand, varies according to the rehabilitation material (more with ceramic than with composite), which is even more pronounced if cortical bone is present than if it is not.

The absence of cortical bone, in peri-implantitis, bone regeneration, or immediate implant placement cases, must be considered when choosing the crown material for the rehabilitation.

## Figures and Tables

**Table 1:** Crown materials and manufactures. Acronyms used in this study.

	Material Name	Manufacturer	Young Modulus [MPa]	Poisson Ratio	Density [g/cm^3^]
**Crown**	**FCOM** *Carbon fiber-composite* BioCarbon Bridge fibers Composite BioXfill	Micro Medica Micro Medica	300,000 22,000	0.3 0.3	1.40 8.30
	**MCER** *Metal-ceramic* Co-Cr alloy Ceramic VMK 95	Renishaw Vita	208,000 69,000	0.31 0.28	8.90 2.50
	**MCOM** *Metal-composite* Co-Cr alloy Composite BioXfill	Renishaw Micro-Medica	208,000 22,000	0.31 0.3	8.90 8.30
	**MET** *Full metal* Co-Cr Alloy, Mo, W	Heraeus Kulzer	208,000	0.31	8.90
	**FCCER** *Carbon fiber-ceramic* Carbon Fiber Bridge Ceramic IPS e.max	Micro-Medica Ivoclar Vivadent	66,000 95,000	0.3 0.2	1.4 2.5
	**PKCOM** *PEEK-composite* PEEK Optima Composite BioXfill	Invibio Micro-Medica	4,100 22,000	0.36 0.3	1.3 8.30
**Implant**	Ti-6-Al-4V ELI	MIS	113,800	0.34	4.43
**Bone**	Cortical bone	[27,28]	15,000	0.3	1.79
	Trabecular bone	[28]	500	0.3	0.45

**Table 2:** Initial and added masses to each model.

Model	Initial Mass (g)	Added Mass (g)
**ET**	8.62	0
**MCER**	6.73	1.89
**MCOM**	6.63	1.99
**FCOM**	5.23	3.39
**PKCOM**	7.20	1.42
**FCCER**	5.07	3.55

**Table 3:** Added mass for both analyses: with and without cortical bone.

Model	Added Mass with Cortical (g)	Added Mass without Cortical (g)
**MET**	0	2.27
**MCER**	1.89	4.16
**MCOM**	1.99	4.26
**FCOM**	3.39	5.66
**PKCOM**	1.42	3.69
**FCCER**	3.55	5.82

**Table 4:** Bone part considered in each simulation.

	Node	Cortical Bone Trabecular Bone	
**Simulation 1**	Trabecular	X	X
	Cortical	X	X
**Simulation 2**	Trabecular		X

**Table 5:** Maximum von Mises stresses obtained in cortical bone node (cortical and trabecular) and trabecular node without cortical bone.

		Maximum Von Mises Stress (MPa)					
Node	Bone	MET	MCER	MCOM	FCOM	PKCOM	FCCER
**Cortical**	trab + cort	63.35	72.06	40.71	35.70	32.05	75.46
**Trabecular**	trab + cort	12.09	15.23	10.30	10.01	9.15	16.69
**Trabecular**	Trab	30.80	33.87	29.30	31.14	22.80	43.33
**trab vs. trab + cort** **∆*σ*_vM_ (%)**		60.75%	55.03%	64.85%	67.85%	59.87%	61.48%

## Data Availability

Not applicable.

## Abbreviations

MET	Metal crown
MCER	Metal-Ceramic crown
MCOM	Metal-Composite crown
FCOM	Carbon fiber-Composite crown
PKCOM	Peek-Composite crown
FCCER	Carbon Fiber-Ceramic crown
FEA	Finite Element Analysis
DIC	Digital Image Correlation
CAD	Computer-Aided Design
CT Scan	Computer Tomography Scanner
